# Sulforaphane-Mediated Nrf2 Activation Prevents Radiation-Induced Skin Injury through Inhibiting the Oxidative-Stress-Activated DNA Damage and NLRP3 Inflammasome

**DOI:** 10.3390/antiox10111850

**Published:** 2021-11-22

**Authors:** Jinlong Wei, Qin Zhao, Yuyu Zhang, Weiyan Shi, Huanhuan Wang, Zhuangzhuang Zheng, Lingbin Meng, Ying Xin, Xin Jiang

**Affiliations:** 1 Jilin Provincial Key Laboratory of Radiation Oncology & Therapy, The First Hospital of Jilin University, Changchun 130021, China; weijl17@mails.jlu.edu.cn (J.W.); jluzhaoqin09@jlu.edu.cn (Q.Z.); zhangyuyu@jlu.edu.cn (Y.Z.); shiwy@jlu.edu.cn (W.S.); wanghh2714@mails.jlu.edu.cn (H.W.); zhengzz2715@mails.jlu.edu.cn (Z.Z.); 2 Department of Radiation Oncology, The First Hospital of Jilin University, Changchun 130021, China; 3 NHC Key Laboratory of Radiobiology, School of Public Health, Jilin University, Changchun 130021, China; 4 Department of Hematology and Medical Oncology, Moffitt Cancer Center, Tampa, FL 33612, USA; lingbin.meng@moffitt.org; 5 Key Laboratory of Pathobiology, Ministry of Education, Jilin University, Changchun 130021, China

**Keywords:** sulforaphane, Nrf2, oxidative stress, NLRP3, radiation-induced skin injury

## Abstract

This article mainly observed the protective effect of sulforaphane (SFN) on radiation-induced skin injury (RISI). In addition, we will discuss the mechanism of SFN’s protection on RISI. The RISI model was established by the irradiation of the left thigh under intravenous anesthesia. Thirty-two C57/BL6 mice were randomly divided into control group (CON), SFN group, irradiation (IR) group, and IR plus SFN (IR/SFN) group. At eight weeks after irradiation, the morphological changes of mouse skin tissues were detected by H&E staining. Then, the oxidative stress and inflammatory response indexes in mouse skin tissues, as well as the expression of Nrf2 and its downstream antioxidant genes, were evaluated by ELISA, real-time PCR, and Western blotting. The H&E staining showed the hyperplasia of fibrous tissue in the mouse dermis and hypodermis of the IR group. Western blotting and ELISA results showed that the inflammasome of NLRP3, caspase-1, and IL-1β, as well as oxidative stress damage indicators ROS, 4-HNE, and 3-NT, in the skin tissues of mice in the IR group were significantly higher than those in the control group (*p* < 0.05). However, the above pathological changes declined sharply after SFN treatment (*p* < 0.05). In addition, the expressions of Nrf2 and its regulated antioxidant enzymes, including CAT and HO-1, were higher in the skin tissues of SFN and IR/SFN groups, but lower in the control and IR groups (*p* < 0.05). SFN may be able to suppress the oxidative stress by upregulating the expression and function of Nrf2, and subsequently inhibiting the activation of NLRP3 inflammasome and DNA damage, so as to prevent and alleviate the RISI.

## 1. Introduction

Radiotherapy (RT) is currently the main treatment for various cancers. While killing tumors, radiation can also bring both short-term and long-term side effects to healthy tissue. Common complications include fatigue, hematological toxicity, skin and mucous membrane damage, and damage to other exposed sites [1]. Radiation-induced skin injury (RISI) is one of the major complications [2]. It has been reported that about 80% of patients with head and neck cancer during RT can present dry or wet dermatitis, mucosal congestion, erosion, ulcers, and other skin mucosal reactions after RT [3]. RISI can affect the dose and tolerance of RT in cancer patients, leading to decreased quality of life and poor tumor control [4,5]. IR can cause DNA strand breaks and a large amount of DNA damage in cells, which can lead to radiation damage [6]. In addition, studies have shown that oxidative stress and inflammatory response may be related to the pathogenesis of RISI [7,8,9].

Inflammasome-activation-mediated inflammation is an important part of the inflammatory response. Nucleotide-binding oligomerization domain-like receptor protein 3 (NLRP3) inflammasome can activate caspase-1, leading to the maturation and release of inflammatory cytokines, including IL-1β and IL-18 [10]. Studies suggested that expression upregulation of NLRP3 inflammasome played an important role in radiation damage, including RISI [11,12]. In addition, previous reports have shown that inflammation can inhibit DNA damage repair responses [13]. Therefore, modulation of targets associated with NLRP3 inflammasomes may serve as a novel therapeutic strategy for RISI.

Studies have shown that the oxidation/reduction (redox) system played a key role in acute radiation-induced injury and regulated a number of effects, such as bystander effects, out-of-field effects, inflammation, and fibrosis [14,15,16]. The overproduction of free radicals and reactive oxygen species (ROS) induced by radiation may give rise to oxidative stress reaction, damage the basal cells of the skin, and prevent the basal cells from division, proliferation, differentiation, and keratinization to induced RISI [17]. In addition, ROS production is the most important trigger for the formation and activation of NLRP3 inflammasome [18]. On the other hand, ROS contribute to the activation of DNA damage and senescence pathways in irradiated cells [19]. Therefore, preventing the production of ROS or increasing the content of endogenous antioxidants is of great significance for the prevention and treatment of RISI [20,21].

Nuclear factor erythroid 2-related factor 2 (Nrf2) is considered to be a very critical transcription factor for antioxidant stress in the body [22]. It can combine with the antioxidant response element in the region of gene promoters, thus inducing the downstream gene transcription of a variety of antioxidative stress/detoxifying enzymes and proteins, such as catalase (CAT), NAD(P)H: quinone oxidoreductase (NQO1), heme oxygenase 1 (HO-1), and superoxide dismutase (SOD), which contribute to the body against oxidative stress and damage [23,24]. Previous studies on the prevention and treatment of oxidative damage by activating the Nrf2 signaling have been reported [25,26]. Therefore, Nrf2 may be an effective target for the prevention of RISI.

Sulforaphane (SFN) is the main hydrolysis product of glucosinolate, which can be extracted from cruciferous vegetables [27]. Previous studies have shown that SFN has multiple biological functions, including anticancer, sterilization, anti-inflammatory, and antioxidative stress [28,29,30]. In addition, many studies on the beneficial effects of SFN after IR have been reported in recent years. SFN can reduce the toxic reaction caused by ionizing radiation and play a protective role [31,32,33]. At present, SFN was recognized as an Nrf2 activator, which indirectly plays an antioxidant role by activating a variety of endogenous antioxidants [34,35]. Our previous experimental results showed that SFN can protect the testis, aorta, and myocardium from diabetes-induced oxidative damage by upregulating Nrf2 function [36,37,38]. However, it is still unclear whether SFN’s activation of Nrf2 can effectively prevent radiation-induced oxidative damage of skin. In addition, it has been recently reported that SFN can selectively suppress activation of the NLRP3 inflammasome to protect pancreatic acinar cell injury [25]. Therefore, in the present study, we investigate whether SFN can prevent RISI and whether its protection is associated with the inhibition of oxidative-stress-mediated DNA damage and NLRP3 inflammasome activation by upregulating Nrf2.

## 2. Methods

### 2.1. Animal Irradiation

Thirty-two 3-month-old C57BL/6 mice were purchased from Beijing Vital River Laboratory Animal Technology Co., Ltd. and placed in the Jilin University Key Laboratory of Pathobiology at 22 °C with a 12-hour light/dark cycle with free water and food. The experimental animals adapted to the laboratory environment 1 week before the experiment. All animal experiments were approved by the Ethics Committee of the First Hospital of Jilin University (approval number: 2020-0869, date: 14 October 2020) and conducted in accordance with national and international (Declaration of Helsinki) guidelines.

The left thigh skin of mice was gently stretched to required dimensions (2.0 cm × 2.0 cm) and exposure for irradiation. Other surrounding body areas were shielded by lead plates. A single dose of 40 Gy 180 kV X-ray was delivered from the X-RAD 320 instrument (Precision X-ray, North Branford, CT, USA) by a dose rate of 200 cGy/min. All experimental mice were randomly assigned to one of the four groups (*n* = 8), including control group (CON), SFN group, irradiation (IR) group, and IR plus SFN (IR/SFN) group. The mice of the SFN group and IR/SFN group were given subcutaneous injection (localization: subcutaneous position on the right inner thigh) of SFN (0.5 mg/kg body weight, Sigma-Aldrich, dissolved in 1% dimethyl sulfoxide and diluted in physiological saline) at 5 days of each week for 1 month. At the same time, the control and IR groups received the same amount of saline containing 1% dimethyl sulfoxide. All mice were kept for an additional 1 month without treatment, euthanized, and skin tissues of the irradiated areas were collected for protein, mRNA, and histopathological analyses.

### 2.2. Mouse Skin Injury Assessment

The body weight, radiation-induced skin injury score, and desquamated wound were observed and recorded in detail at three time cut-off points in the first week (1 w), the fourth week (4 w), and the eighth week (8 w) after irradiation. RISI scores were evaluated by a radiation-induced mouse skin injury scoring criteria, which is slightly different from the previous grading scale [39] (Table 1). Desquamated wound was measured by digital camera with a high resolution ratio.

### 2.3. Pathological Examination

The skin tissue was fixed in formaldehyde (10%) for 1 day and then embedded with paraffin. The section thickness was 5 µm. Then, skin paraffin was dewaxed and hydrated. Finally, it was dyed with hematoxylin and eosin (H&E). The pathological changes were observed under a microscope.

### 2.4. RNA Isolation and Real-Time PCR

Real-time PCR was used to analyze NLRP3 inflammasome, as well as Nrf2 downstream genes of HO-1 and CAT, in skin tissues. RNA was extracted from mice skin tissue using prepared Trizol reagent. We then synthesized cDNA using random primers. Then, Applied Biosystem SPRISM 7700 quantitative PCR instrument and specific primer sequences were used for real-time PCR analysis. The primer sequences were purchased by TransGen Biotech Co., Ltd (Beijing, China). All primer sequences are shown below: GAPDH, 5′-GGTGAAGCAGGCGTCGGAGG-3′ and 5′-GAGGGCAATGCCAGCCCAG-3′; NLRP3, 5′-GTGGAGATCCTAGGTTTCTCTG-3′ and 5′-CAGGATCTCATTCTCTTGGATC-3′; caspase1, 5′-ACACGTCTTGCCCTCATTATCT-3′ and 5′-ATAACCTTGGGCTTGTCTTTCA-3′; IL-1β, 5′-CCCTGCAGCTGGAGAGTGTGG-3′ and 5′-TGTGCTCTGCTTGAGAGGTGC-3′; HO-1, 5′-ATGGCCTCCCTGTACCACATC-3′ and 5′-TGTTGCGCTCAATCTCCTCCT-3′; CAT, 5′-TGAAGATGCGGCGAGACTTT-3′ and 5′-TGGATGTAAAAAGTCCAGGAGGG-3′.

### 2.5. Western Blotting

Skin tissue was lysed in RIPA lysis buffer and protein was collected under centrifugation at 12,000× *g* rpm for 15 min at 4 °C. Protein concentrations in skin tissues were measured. The protein sample was diluted with loading buffer and boiled at 100 °C for 5 min. The protein samples were then separated by SDS-PAGE (concentration: 10%). They were then transferred to the PVDF membrane. The membrane was sealed with blocking buffer (5% milk) for 1 h, and incubated overnight with primary antibodies in a 4 °C refrigerator. The primary antibodies included NLRP3, caspase-1, IL-1β, γH2AX, Nrf2 (1:1000, Affinity Biosciences, Cincinnati, OH, USA), 3-NT (1:1000, Millipore, Boston, MA, USA), 4-HNE (1:1000, Alpha Diagnostic International, San Antonio, TX, USA), HO-1, CAT, and anti-β-actin (1:1000, Santa Cruz Biotechnology, Santa Cruz, CA, USA). The membrane was then rinsed with TBST (0.05% Tween 20) three times and incubated with horseradish peroxidase-labeled secondary antibody for 1 h at room temperature. Finally, ECL kit was used to indirectly detect protein expression level. All experiments were repeated at least three times. Image J v1.8.0 software was used to conduct grayscale analysis of protein expression bands.

### 2.6. Enzyme-Linked Immunosorbent Assay (ELISA)

The serum of mice was collected. The operation was carried out according to the operation instructions of the mouse ROS ELISA kit and 8-OhdG ELISA kit purchased from Shanghai Enzyme-Linked Biotechnology Co., LTD. The standard substance and the sample to be tested were added to the pre-encapsulated polyclonal antibody transparent enzyme-labeled plate. Then, we added the enzymatic working solution and sealed it with a membrane. After incubation for 60 min, we washed and removed the unbound ingredients. Substrates A and B were added in order to determine the absorbance value at the wavelength of 450 nm. The concentrations of ROS and 8-OHDG in the serum of mice were calculated according to the absorbance values of the standard substance and the sample.

### 2.7. Statistical Analysis

After completing the experiment, we used IBM SPSS Statistics for Windows, Version 24.0, to collect and analyze all data. Data were expressed as mean ± standard deviation. One-way ANOVA was used for comparison of multiple groups. At the same time, the Tukey’s test was adopted for comparison of two groups. Statistical results with *p* < 0.05 were deemed to have statistical difference.

## 3. Results

### 3.1. SFN Decreases the RISI in Mice

**Figure 1:**
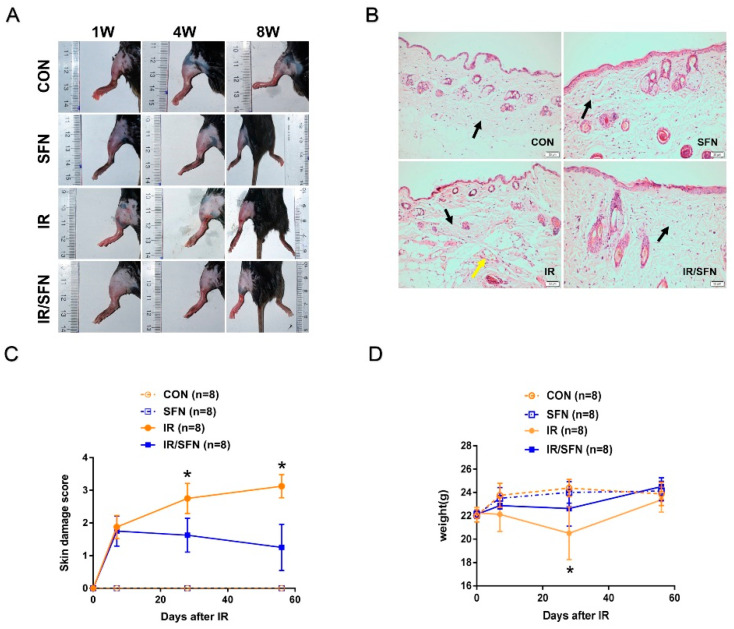
Results of skin injury and repair in mice. (**A**): Photographs of skin wound in 4 groups of mice at week 1, week 4, and week 8 after irradiation exposure. Scale bars = 4 cm. (**B**): H&E staining results of mouse skin tissue. Black arrows represent fibrous connective tissue. Yellow arrows represent proliferating blood vessels. (H&E staining, original magnifications × 200, bar = 50 μm.) (**C**): Skin damage scores of mice at week 1, week 4, and week 8 after irradiation exposure were expressed as the mean ± S.D. (**D**): Mean weights ± S.D. of 4 groups of mice at week 1, week 4, and week 8 after irradiation exposure (*n* = 3 at least in each group) (* *p* < 0.05 vs. CON).

In the IR group, the skin damage was gradually aggravated after initial irradiation. The mice skin showed generalized erythema at 1 w, obvious erythema and moist desquamation at 4 w, and patchy moist desquamation with local ulcers at 8 w (Figure 1A). However, in the IR/SFN group, mice skin only showed a small amount of scattered erythema in 1 w, 4 w, and 8 w (Figure 1A).

The H&E staining results showed hyperplasia of fibrous tissue, irregular arrangement of collagen fiber bundles, and proliferating blood vessels in the mouse dermis and hypodermis of the IR group at 8 w. These pathological changes were not detected in the unexposed group, including the control group and SFN group. In the IR/SFN group, only fibrous tissue hyperplasia was observed, and other pathological changes were not obvious. In addition, the hyperplasia of fibrous tissue was less than that of the IR group (Figure 1B).

Mouse skin damage scores of the IR/SFN group were lower than the IR group at 4 w and 8 w after irradiation (*p* < 0.05) (Figure 1C). Compared with the control group, the body weight of mice in the IR group and IR/SFN group was decreased at 4 w and recovered to normal at 8 w. Meanwhile, the body weight of IR/SFN group mice was significantly higher than IR group mice (*p* < 0.05) (Figure 1D).

### 3.2. SFN Prevents Radiation-Induced DNA Damage in Skin

**Figure 2:**
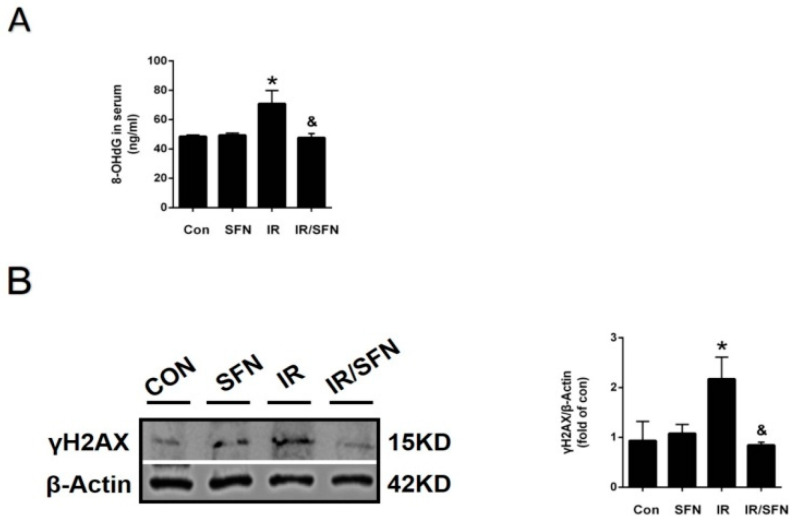
SFN prevents DNA damage caused by radiation. (**A**): The expression of 8-OHdG in skin blood serum was examined by ELISA. (**B**): The expression of γ-H2AX in skin tissues was examined by Western blotting. Data were expressed as the mean ± S.D. (*n* = 3 at least in each group) (* *p* < 0.05 vs. CON; ^&^ *p* < 0.05 vs. IR).

The level of 8-OHdG in mouse serum was determined by ELISA, and the expression of γH2AX, a DNA damage indicator in skin tissues, was detected by Western blotting. 8-OHdG production and γH2AX expression in the IR group were significantly increased compared to those in the control group. However, compared with the IR group, they were significantly decreased in the IR/SFN group (Figure 2A,B).

### 3.3. SFN Prevents Radiation-Induced Activation of NLRP3 Inflammasome in Skin

**Figure 3:**
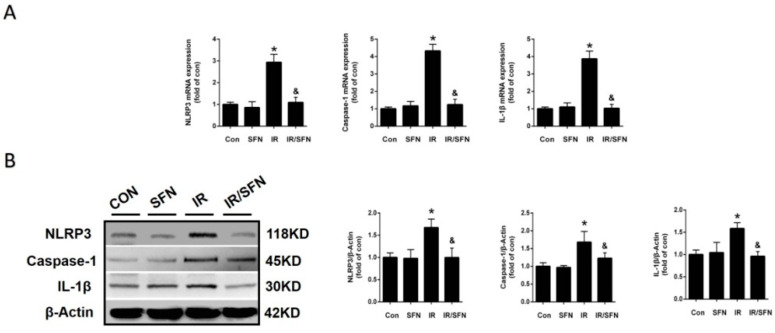
Prevention and treatment of SFN on radiation-induced skin inflammation. (**A**): The expressions of inflammatory factors, including NLRP3, caspase-1, and IL-1β mRNAs, were examined by real-time PCR. (**B**): The protein expressions of inflammatory factors, including NLRP3, caspase-1, and IL-1β, in skin tissues were examined by Western blotting. Data were expressed as the mean ± S.D. (*n* = 3 at least in each group) (* *p* < 0.05 vs. CON; ^&^ *p* < 0.05 vs. IR).

Studies have shown that activation of NLRP3 inflammasome played an important role in radiation damage, including RISI [12]. Therefore, the expression of NLRP3, caspase-1, and IL-1β in skin tissues were evaluated by real-time PCR and Western blotting. Compared with the control group, the expression of these inflammatory factors in the IR group was significantly increased at both mRNA and protein levels, which was almost completely prevented by SFN treatment (Figure 3A,B).

### 3.4. SFN Prevents Radiation-Induced Oxidative Stress in Skin

**Figure 4:**
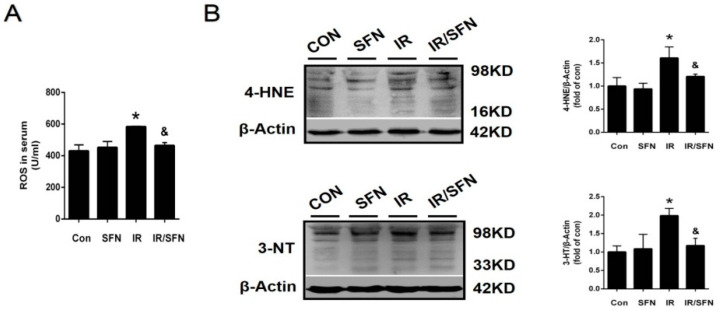
Prevention and treatment of SFN on radiation-induced skin oxidative stress. (**A**): The expression of ROS in serum was examined by ELISA. (**B**): The expressions of 3-NT and 4-HNE were measured by Western blotting due to the oxidative damage in the skin tissues. Data were expressed as the mean ± S.D. (*n* = 3 at least in each group) (* *p* < 0.05 vs. CON; ^&^ *p* < 0.05 vs. IR).

The expression of ROS in serum was determined by ELISA. Compared with the control group, IR treatment induced an elevation in ROS level in the mouse serum, an effect that is prevented by SFN treatment. (Figure 4A). At the same time, intervention of IR also induced the accumulation of 4-HNE (an index of lipid peroxidation) and 3-NT (an index of nitrosative damage) in the skin tissue, which was almost completely prevented by SFN treatment (Figure 4B).

### 3.5. SFN Upregulated the Expression and Function of Nrf2 in Skin

Studies have confirmed that SFN can upregulate the expression and function of Nrf2, and Nrf2 is considered to be the main regulator of redox status and cellular detoxification responses [40]. Therefore, the preventive effect of SFN on RISI by activating Nrf2 was detected by the expression of Nrf2 and its transcriptional function in skin.

**Figure 5:**
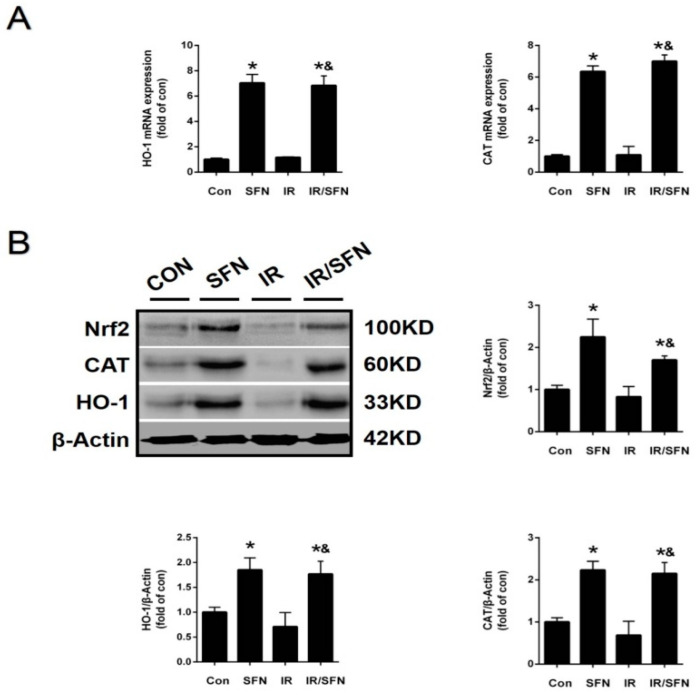
Activation of Nrf2 and expressions of its downstream genes in the skin induced by SFN. (**A**): The Nrf2 transcription function was examined by the expression of its downstream antioxidant gene (CAT, HO-1) mRNAs by real-time PCR. (**B**): The expression of Nrf2 in mouse skin tissues was detected by Western blotting. Nrf2 transcription function was examined by the expressions of its downstream antioxidant (CAT, HO-1) proteins with Western blotting. Data were expressed as the mean ± S.D. (*n* = 3 at least in each group) (* *p* < 0.05 vs. CON; ^&^ *p* < 0.05 vs. IR).

Western blotting results confirmed that the expression level of Nrf2 in the skin tissue was increased in SFN group. IR treatment did not significantly affect skin Nrf2 expression. However, compared with the IR group, the Nrf2 expression was increased in the IR/SFN group (Figure 5A,B). At the same time, Nrf2 function was confirmed by the expression of its downstream antioxidant enzymes, including HO-1 and CAT, by real-time PCR and Western blotting. Compared with the control group, the mRNA and protein expression of CAT and HO-1 was increased in the SFN group and IR/SFN group, but not in the IR group. Furthermore, the expression of these enzymes in the IR/SFN group was higher than the IR group (Figure 5A,B). 

## 4. Discussion

The characteristic of RISI is a complication of present dry or wet dermatitis, mucosal congestion, erosion, and ulcers caused by irradiation [41,42]. There is increasing evidence that oxidative stress and inflammatory response may be related to the pathogenesis of RISI [4,8]. Therefore, it is promising to find a new therapeutic strategy that focuses on both anti-inflammatory and antioxidant effects, particularly in the early stages of RISI, to prevent the development of more severe skin lesions. SFN, as a highly effective phytochemical antioxidant, has not been fully evaluated on the radiation-induced skin lesions [37,43].

SFN, which is extracted from cruciferous vegetables, has a powerful anticancer effect, as well as anti-inflammatory and antioxidant effects [44,45]. In previous studies, SFN has been used as an antioxidant for treatment ranging a few hours in vitro to a week or weeks in vivo [45,46]. Our previous experimental results showed that SFN played an antioxidative stress role, thus protecting against testicular injury caused by diabetes [36]. In our study, the RISI of mice in the IR/SFN group treated by SFN was better than the IR group at each time point of skin damage evaluation, indicating that SFN played a role in alleviating RISI (Figure 1A,C). Subsequently, we verified the skin pathological changes of mice after radiation by H&E staining. The IR group showed significant changes, including fibrous tissue hyperplasia, irregular arrangement of collagen fiber bundles, and proliferating blood vessels. However, in the IR/SFN group, only a little fibrous tissue hyperplasia was observed, and other pathological changes were not obvious (Figure 1B). It is also confirmed that SFN can reduce the effect of RISI.

In recent years, NLRP3 inflammasome has become an important molecule in the pathogenesis of RISI [11]. It has been previously reported that SFN can inhibit caspase-1 activation and IL-1β maturation and secretion, which is a downstream of NLRP1 and NLRP3 in immune cells through an Nrf2-independent mechanism [47]. Our data showed that the activation of NLRP inflammasome is accompanied with RISI, and SFN treatment significantly inhibited the upregulation of those inflammatory factors NLRP3, caspase-1, and IL-1β (Figure 3A,B). This demonstrated that SFN can inhibit the activation of NLRP3 inflammasome, playing a strong anti-inflammatory role, to prevent RISI. In addition, we found that the degree of DNA damage in mouse skin, in accordance with NLRP3 inflammasome activation, was significantly reduced with SFN treatment (Figure 2A,B). This proves that SFN may play a role in alleviating IR-induced DNA damage by preventing NLRP3-mediated inflammation. At the same time, the reduction in DNA damage may also decrease the activation of NLRP3 inflammasome.

Oxidative stress referred to the imbalance between the produce of ROS and RNS and the clearance of these free radical byproducts [48]. More evidence has shown that oxidative stress is involved in the occurrence and development of various diseases, including RISI [14,49]. Studies in vitro suggest that oxidative stress can play a key role in the pathogenesis of radiation damage [50]. Consistent with this, irradiation also induced the oxidative damage to mouse skin, reflected by the increased accumulations of ROS, 3-NT, and 4-HNE. However, ELISA and Western blotting confirmed that SFN could inhibit the expression of these oxidative stress indicators and serum level of ROS (Figure 4A,B). Meanwhile, along with the decrease in ROS, the activation of NLRP3 inflammasome was also inhibited. This is consistent with the understanding that ROS is an activator of NLRP3 inflammasome.

Nrf2, as a major endogenous antioxidant defense system, played an important role in the prevention of oxidative damage [35]. CAT and HO-1 are very crucial downstream antioxidant genes in the Nrf2 signaling pathway. In addition, study has confirmed that the activation of Nrf2 and its downstream genes can protect against diabetes-induced organ injury [51]. However, previous studies on the prevention of radiation-induced oxidative stress damage by SFN-mediated Nrf2 activation have not been reported. Here, we have proved that SFN can increase the expression and function of Nrf2 in CON group l mouse skin and IR skin, evidenced by the higher expression of Nrf2 and its downstream of CAT and HO-1 (Figure 5A,B). More importantly, the skin Nrf2 activation by SFN was accompanied with the prevention of skin damage caused by IR-induced oxidative stress, which indicated that the protective effect of SFN on RISI was closely related to the upregulation and activation of skin Nrf2.

In our study, the most innovative discovery was that SFN provides skin protection from IR. RISI in clinical patients during RT have been a problem in the field. At present, there are a few drugs to treat RISI in clinic, but the effect is not very ideal or some may cause certain side effects. However, SFN, extracted from natural broccoli, has no toxicity and is easily accepted for usage in clinic. According to our findings, SFN will provide a new strategy for clinical treatment and prevention of RISI in the future.

## 5. Conclusions

**Figure 6:**
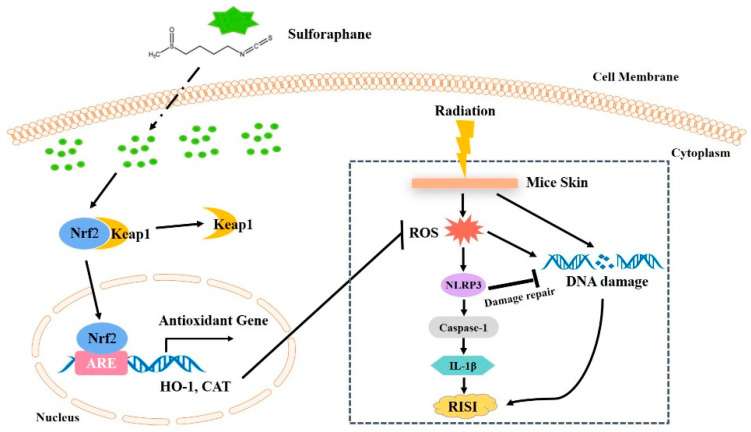
Illustration of the mechanism of radiation-induced skin injury prevented and treated by SFN. SFN can upregulate antioxidant enzymes, including HO-1 and CAT, through the activation of Nrf2 and inhibit the NLRP3 inflammatory pathways by suppressing the ROS production, alleviating radiation-induced skin injury (RISI) in mice. In addition, inhibition of NLRP3 inflammasome may have a positive effect on DNA damage repair, thereby promoting the repair of RISI.

In conclusion, our study demonstrated that SFN can upregulate antioxidant enzymes, including HO-1 and CAT, through the activation of Nrf2 and inhibit the NLRP3 inflammasome by suppressing the ROS production, alleviating RISI in mice. In addition, inhibition of NLRP3 inflammasome may have a positive effect on DNA damage, thereby promoting the repair of RISI (Figure 6). Therefore, the broccoli extract SFN has a positive modulatory effect on RISI by inhibiting Nrf2-mediated oxidative stress, as well as NLRP3 inflammasome. In the future, oral administration of natural broccoli extract sulforaphane (SFN) may be a new strategy for the prevention of RISI in cancer patients.

## Figures and Tables

**Table 1:** Radiation-induced mouse skin injury scoring criteria.

Score	Manifestation
0.00	Normal
0.50	Very slight reddening
1.00	Severe reddening
1.50	Moist breakdown in one very small area with scaly or crusty appearance
2.00	Moist desquamation in a few areas
2.50	Moist desquamation in half of the irradiated area
3.00	Moist desquamation in most of the irradiated area with possible slight moist exudates
3.50	Moist desquamation of the irradiated area with moist exudates, necrosis

Adapted from Douglas & Fowler, 2012 [39].

## Data Availability

All data generated or analysed during this study are included in this published article. The datasets used or analysed during the current study are available from the corresponding author on reasonable request.

## Abbreviations

CAT	catalase
ELISA	enzyme-linked immunosorbent assay
H&E	hematoxylin and eosin
HO-1	heme oxygenase 1
IR	irradiation
NLRP3	nucleotide-binding oligomerization domain-like receptor protein 3
NQO1	NAD(P)H qui-none oxidoreductase
Nrf2	nuclear factor erythroid 2-related factor 2
RT	radiotherapy
RISI	radiation-induced skin injury
ROS	reactive oxygen species
SFN	sulforaphane
SOD	superoxide dismutase
